# Laparoscopic Salpingectomy and Vasectomy to Inhibit Fertility in Free-Ranging Nutrias (*Myocastor coypus*)

**DOI:** 10.3390/ani13061092

**Published:** 2023-03-19

**Authors:** Giuseppe Bonaffini, Matteo Serpieri, Chiara Ottino, Luca Scandone, Giuseppe Quaranta, Mitzy Mauthe von Degerfeld

**Affiliations:** Centro Animali Non Convenzionali—Dipartimento di Scienze Veterinarie, Università degli Studi di Torino, Largo Paolo Braccini 2, 10095 Grugliasco, Italy; giuseppe.bonaffini@unito.it (G.B.); matteo.serpieri@unito.it (M.S.); chiara.ottino@unito.it (C.O.); luca.scandone@unito.it (L.S.); giuseppe.quaranta@unito.it (G.Q.)

**Keywords:** *Myocastor coypus*, population control, infertilization, salpingectomy, vasectomy, laparoscopic surgery, wild animals

## Abstract

**Simple Summary:**

The nutria (*Myocastor coypus*), an invasive alien species widely spread in Europe is considered a pest in many regions. Several containment plans have been proposed and, often, euthanasia is the only available option. An alternative approach, based on surgical reproduction control of nutrias is evaluated in the present study. Laparoscopic salpingectomy and vasectomy make it possible to inhibit fertility with prompt recovery and release of the animals back into nature. This technique could be applied in urban and anthropized areas, where application of other control methods could be hindered by public opinion. The outcome of the developed and evaluated procedures suggests that laparoscopic salpingectomy and vasectomy represent an effective, safe, and versatile tool for the management of invasive alien species.

**Abstract:**

The nutria (*Myocastor coypus*), an invasive alien species, is widely spread in Europe. Pursuant to regulation (EU) no. 1143/2014, the nutria is subject to management programs to reduce its spread. Surgical fertility control is considered an acceptable method, particularly in urban circumstances, avoiding euthanasia. To maintain the hormonal patterns and the social and behavioral dynamics, surgical infertilization preserving the gonads (i.e., salpingectomy and vasectomy) is recommended. Mini-invasive surgery is an eligible choice when dealing with wildlife, allowing reduced captivation time. For these reasons, 77 free-ranging nutrias, captured in urban nuclei in Italy, underwent infertilization under general anesthesia; laparoscopic salpingectomy and vasectomy were performed on 32 animals and traditional surgery on the remainder, leaving the gonads in place. A three-port technique was used, with two paramedian trocars (5 mm) for the instruments and a median one for the telescope. Ablation was obtained through Onemytis^®^ plasma device, allowing a rapid surgical time with no need to place visceral sutures; the skin was surgically closed. After recovery, the animals were released, and no overt complications were noted. No modification of the behavioral patterns was noted, and the population decreased during the following months.

## 1. Introduction

The nutria (*Myocastor coypus*) is a semi-aquatic rodent, native to South America, widespread throughout the world and highly bred in the past to produce meat and fur. Following the collapse of the tanning industry, many animals were released into the wild and, due to their great adaptability and in the absence of specific predators, they have settled and reproduced so widely that they were included in the list of “invasive alien species of Union concern” by Commission Implementing Regulation (EU) no. 2016/1141. Pursuant to regulation (EU) no. 1143/2014 and, in Italy, to D.L. 230/17, concerning management of invasive alien species (IAS), a national plan for the management of the species, aimed at its monitoring, containment or eradication was put into action.

The nutria is considered a pest in many regions because of its potentially severe effects on biodiversity, economy, ecosystem functionality, and public health [1,2]. Therefore, this rodent has been the object of research projects aimed mainly at defining its distribution, habits, and impacts on the environment. In Italy, regional population containment plans—aimed at mitigating economic damage and safeguarding the territory and biodiversity [3,4]—have been put into action with relative success without, however, permanently solving the issue, above all because the cleared areas are rapidly re-colonized by other subjects. Furthermore, these projects—which aim at simultaneous culling of all the animals in a designated area—meet with resistance from public opinion [5]; moreover, their implementation is difficult to achieve when animals settle in urban and anthropized areas. From this perspective, the control of the population in delimited areas through sterilization appears to be a more acceptable method and, in specific situations, is the only feasible one [6,7,8]. In this alternative approach, it is essential to evaluate the measures to be undertaken and to carry out an analysis of the costs, the procedures (in compliance with the current legislation), the sustainability, the effectiveness, and the efficiency of the available measures [6]. Thus, this protocol ensures that the animals are captured, sterilized, and released back into the wild, allowing the resident subjects to continue to protect resources and prevent the colonization of their habitat by new individuals. However, when dealing with IAS, the legislation obliges the procedures to be carried out directly under field conditions, to avoid the return of the animals in their habitat after the procedures being legally interpreted as a new release into nature.

The standard surgical techniques for reproductive control (ovariectomy and orchiectomy) are not to be considered elective on wildlife, as they modify the hormonal pattern and, consequently, the sexual behaviors and the population dynamics. Salpingectomy and vasectomy—in which salpinges or vasa deferentia are interrupted to induce infertility—prevent contact between gametes while maintaining hormones production [9,10,11,12,13,14]. Similar procedures have been successfully performed in several species (wolves, coyotes, and drills), and no changes in social behaviors has been noted, suggesting the preservation of the normal hormonal pattern [10,11,12,13,14,15].

Salpingectomy and vasectomy can be performed using traditional surgical techniques or with minimally invasive surgery (MIS), as already described for control of animal reproduction in various species, such as wild boars, lions, macaques, and dogs [16,17,18,19,20]. However, the application of innovative techniques—although tested on some non-domestic species—must be carefully evaluated before being suggested as routinely suitable on a new species, considering anatomical, biological, and physiological peculiarities [21].

The purpose of the present study is to evaluate the efficacy of laparoscopic salpingectomy and vasectomy techniques performed on groups of nutrias (*Myocastor coypus*) living in confined urban environments, considering possible surgical and anesthetic complications, as bibliographic reports relating to this technique applied on this species are lacking.

## 2. Materials and Methods

### 2.1. Animal Welfare

Ethical approval was given by the Departmental Executive Committee of the Veterinary Science Department of University of Turin (report N. 2/2018 e N. 4/2021). The study was conducted from February 2018 to March 2022.

### 2.2. Inclusion Criteria

In the context of projects with the Metropolitan City of Turin (CMT) and with the Municipality of Sesto San Giovanni (MI), 77 free-ranging nutrias from confined urban areas were captured. Each subject, once captured and anesthetized (the procedures regarding capture and anesthesia, for all animals, are explained in Section 2.4) underwent abdominal ultrasound to evaluate their reproductive tract status. Pregnant animals, subjects weighing less than 1000 g and subjects in which ultrasound showed an excessively replete intestinal tract or pathologic reproductive tract, were assigned to the Traditional group (Group T; *n* = 44); on these animals, hysterectomy, salpingectomy, or vasectomy via laparotomy was performed. The remaining subjects were assigned to the Laparoscopy group (Group L; *n* = 33), where laparoscopic salpingectomy and vasectomy were performe.

One nutria with a large wound on its back and severe myiasis was euthanized.

### 2.3. Mobile Field Unit

Complying with D.L. 230/17, the afternoon before the day of the procedures, a temporary field unit was set up. The unit consisted of two folding gazebos (FleXtents PRO, Dancover A/S, Hillerød, Denmark): one intended for hospitalization, induction, and recovery from anesthesia, before and after surgery; the other one for the clinical examination, biometric measurements, and pre-surgical patient preparation. A specifically equipped mobile field unit (Fiat Ducato, 2007, Turin, Italy) of the Department of Veterinary Sciences of the University of Turin—set up as a mobile surgical theater—was positioned alongside the gazebos.

### 2.4. Capture, Anesthesia and Preparation

The nutrias were captured with cage traps (Live animal trap, SGD Group Srl., Treviso, Italia) filled with vegetables, regularly checked by trained technicians. Once captured, the animals were kept for a minimum of 2 h in a sheltered gazebo to reduce the stress levels. The animals underwent surgery following the capture line-up, with the purpose of reducing the length of captivation as much as possible.

The nutrias were anesthetized following the procedures described by Mauthe et al. [22], injecting 6 mg/kg of ketamine (Ketavet 100, 100 mg/mL, Intervet Productions Srl, Aprilia, Italy) and 0,14 mg/kg of medetomidine (Sedator, 1 mg/mL, Dechra Veterinary Products Srl, Turin, Italy), into the thigh muscles, through the mesh of the cages.

A minimum trichotomy and abdominal ultrasound scan (for purposes described at point 2.2) were then performed. Subcutaneous carprofen (4 mg/kg, Rimadyl Iniettabile, 50 mg/mL, Zoetis Italia Srl, Rome, Italy) and lukewarm fluids (30 mL/kg, 1:1 mixture of Ringer’s lactate and 5% glucose; both Baxter SpA, Rome, Italy) were administered, and the animals were then brought into the mobile unit for the surgical procedures (described in Section 2.5).

Intraoperative anesthetic monitoring was performed using a multiparametric monitoring system (Infinity Delta, Dräger Italia SpA, Corsico, Italy). Vital parameters were measured through electrocardiography (ECG) and pulse-oximetry; the pulse-oximeter probe was placed on the pinna or on the foot. Pure oxygen was given through a face mask (anesthetic face mask, M, Jørgen Kruuse A/S, Langeskov, Denmark) to each animal.

In case of a 20% increase in HR, isoflurane (IsoFlo 100%, Zoetis, Rome, Italy) was dispensed using a vaporizer (Datex Ohmeda Tec 5, Soma Technology Inc., Bloomfield, Connecticut, USA) and delivered through a rebreathing system, to maintain an adequate anesthetic depth. Manual assisted ventilation at 12 breaths/min was required for rodents undergoing minimally invasive surgery.

Once surgical procedures were completed, the nutrias were moved back to the gazebo and atipamezole hydrochloride (Sedastop, 5 mg/mL, Ecuphar Italia Srl, Milan, Italy) was administered intramuscularly at a dosage 2.5 times higher than that of medetomidine.

Subsequently, a depot antimicrobial (0.2 mL/kg of a combination of benzathine benzylpenicillin and dihydrostreptomycin sulphate; Rubrocillina Forte Veterinaria, 250,000 IU/mL + 100 mg/mL, Intervet Productions Srl, Aprilia, Italy) was administered subcutaneously, due to the impracticability of treating the animals after release.

The animals were monitored until complete recovery (all reflexes reappeared and spontaneous movements after administration of the antagonist).

A transponder tag was inserted in the left prescapular area (Therachip^®^, Bioforlife Italia S.r.l., Milan, Italy), as requested by CMT and the Municipality of Sesto San Giovanni.

Once on the mobile unit, the nutrias were placed in dorsal recumbency and the ventral surface of the abdomen was aseptically prepared with 10% povidone–iodine solution (Betadine^®^, Meda Pharma, Milan, Italy). The patient was then covered with a sterile drape (sterile surgical drape, 75 × 90 cm, Medline Industries Inc., Northfield, MN, USA.) and tilted in Trendelenburg position (surgical table tilted approximately 30 degrees, with head down) to cranially displace the abdominal viscera.

### 2.5. Surgical Procedure—Group L

In this group, *n* = 32 nutrias of various age, sex and weight were included. The ventral abdominal wall was palpated to locate and displace the spleen towards the left. A skin incision 1 cm caudal to the umbilicus, by use of a No. 11 scalpel blade, was made; the abdominal wall was lifted upwards and a Veress needle (Veress, Ø 2,1 mm, length 7 cm, Karl Storz GMBH & Co. KG, Tuttlingen, Germany) was inserted. A 5 mL syringe was connected to it and negative pressure was applied to check for possible presence of blood or enteric material. In the absence of content, a sterile tube connected to an automatic insufflator (Electronic Laparoflator^®^, Karl Storz GMBH & Co. KG, Tuttlingen, Germany) was connected to the Veress needle.

The abdominal cavity was insufflated with CO_2_ at a flow rate of 1 L/min up to a pressure of 14 mmHg. After insufflation, the needle was removed and a trocar (Trocar System, Ø 5.5 mm, Vomed Volzer Medizintechnik GmbH & Co. KG., Tuttlingen, Germany) was inserted in its place. The insufflating tube was connected to it to maintain a constant pressure of 14 mmHg. A laparoscope with 30° viewing angle (Olympus model A50372A 30°, Ø 5 mm, Olympus Europa SE & Co. KG., Hamburg, Germany), connected to a light source (Ubilight^®^, 100 W LED lamp, Sopro SA, La Ciotat, France) and a video camera (Ubicam^®^, USB camera, Sopro SA, La Ciotat, France), was inserted through the trocar.

Prior to placing the additional operating ports, the laparoscope light source was used to transilluminate the ventral abdominal wall and identify the subcutaneous blood vessels. Two skin incisions were made in areas without large vessels, paramedian and cranial to the first incision for females and paramedian and caudal for males, following the standard procedure for laparoscopic triangulation.

Two operating trocars were inserted through the incisions (Trocar System, Ø 5.5 mm, Vomed Volzer Medizintechnik GmbH & Co. KG, Tuttlingen, Germany), under endoscopic vision to avoid damaging the viscera. The CO2 insufflation pressure was then reduced and maintained to 8 mmHg.

#### 2.5.1. Laparoscopic Salpingectomy

A fenestrated grasper (Johann grasper, Ø 5 mm, 36 cm, Multimage Srl., Varese, Italy) was inserted through the right paramedian port and was used to retract the intestinal loops, thus allowing visualization of the right ovary, salpinx, and ovarian ligaments. A Babcock forceps (Click Line Babcock grasping forceps, Ø 5 mm, 36 cm, Karl Storz GMBH & Co.), inserted through the left paramedian trocar, was used to grasp to grasp the right salpinx.

**Figure 1:**
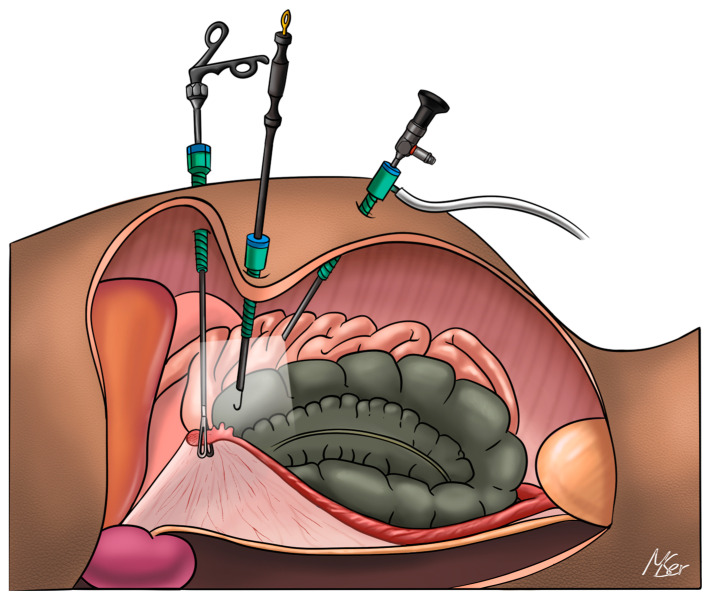
Instruments positioning for salpingectomy in *Myocastor coypus* (right lateral view).

The fenestrated grasper was removed and replaced with a hook (monopolar hook, Ø 5 mm, 36 cm, Otech Industry S.r.l., Alessandria, Italy) connected to an electrosurgical device (Onemytis^®^, Otech Industry S.r.l., Alessandria, Italy) using Airplasma^®^ technology (Figure 1). The hook was used to seal and cut the salpinx. The contralateral salpinx was cut with the same procedure.

#### 2.5.2. Laparoscopic Vasectomy

**Figure 2:**
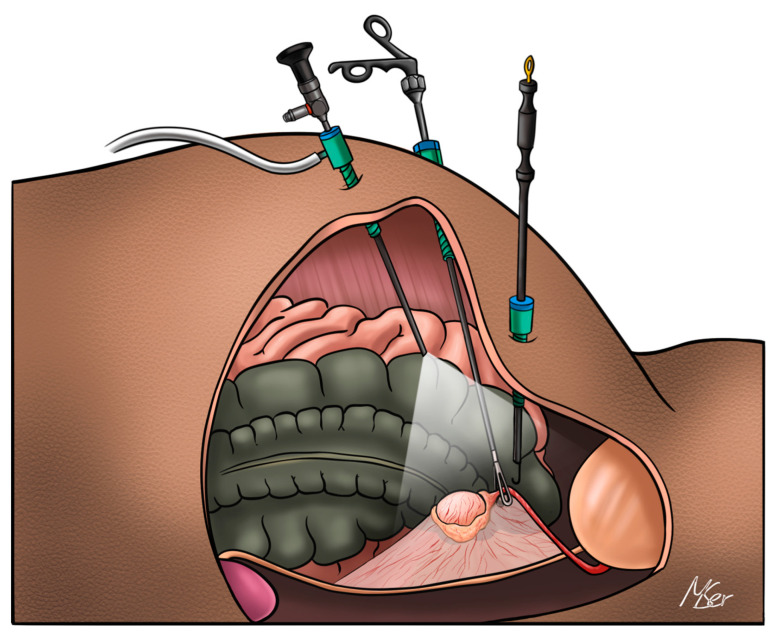
Instrument positioning for vasectomy in *Myocastor coypus* (right lateral view).

The laparoscope was ventrally and caudolaterally directed in search of the right testis. A fenestrated grasper, inserted into the left paramedian trocar, was used to retract the intestinal loops. Once the testis was identified, the vas deferens was grasped. The hook was inserted through the right paramedian port and used to cauterize and cut the right vas deferens (Figure 2). The cut ends of the vas deferens were inspected to verify any bleeding. The same procedure was repeated for the contralateral vas deferens.

In all subjects, upon completion of the procedures, the abdominal cavity was rapidly inspected to highlight any surgery-related trauma. The paramedian trocars were removed under laparoscopic vision. The laparoscope and its trocar were removed, and external pressure was applied to each side of the abdominal wall to facilitate the leakage of the insufflated CO_2_ through the incisions.

Incisions were closed in two layers. The muscular layer was sutured with a simple, interrupted 2-0 absorbable monofilament suture (PDS II™ Ethicon Inc, Milan, Italy) The subcutis and skin were stitched with a continuous intradermal pattern, using a 2-0 absorbable multifilament suture (Vicryl™ Ethicon Inc, Milan, Italy) with sunken knots.

### 2.6. Surgical Procedure—Group T

In this group, *n* = 44 nutrias of various age, sex and weight were included.

#### 2.6.1. Laparotomy

A 5 cm incision was made through the skin and subcutaneous tissues to expose the linea alba, using a No. 21 scalpel blade, 2 cm caudal to the umbilicus. The abdominal wall was stretched upwards with Adson-brown forceps and an incision on linea alba was made. The incision was extended cranially and caudally with dissecting scissors.

#### 2.6.2. Laparotomic Salpingectomy

The apex of the right uterus was manually exteriorized through the incision. The salpinx was then identified, sealed, and cut using a handpiece (Otech Industry S.r.l., Alessandria, Italy) connected to an electrosurgical device (Onemytis^®^, Otech Industry S.r.l, Alessandria, Italy), bilaterally.

#### 2.6.3. Laparotomic Vasectomy

The right testis was manually exteriorized through the incision. The vas deferens was identified, sealed, and cut using the same handpiece used for salpingectomy, bilaterally.

#### 2.6.4. Hysterectomy

Both uteri were removed, while ovaries were left in situ. The right ovary was manually exteriorized through the incision and the ovarian pedicle was identified. A hemostatic forceps was applied caudal to it, near the tip of the right uterus. On the ovarian pedicle, absorbable suture material (PDS II™, 2-0, Ethicon Inc, Milan, Italy) was applied to close the ovarian branches of the uterine artery, cranial to the hemostatic forceps. The ovarian pedicle and the mesovarium were dissected between the forceps and the suture. The same procedure was performed on the other side. Subsequently, the uteri were exteriorized, and cranial traction was applied to them. A hemostatic forceps was applied cranial to the cervix. Between the hemostatic forceps and the cervix, a figure-of-eight suture was applied, piercing the most caudal portions of the uteri; also, a circumferential suture around the uteri was applied caudally. Absorbable suture material (PDS II™, 2-0, Ethicon Inc, Milan, Italy) was used. The most caudal portions of the uteri were incised between the hemostat and the ligatures, and the uteri could be extracted.

#### 2.6.5. Closure of Surgical Incisions

Finally, the abdominal wall was sutured in two layers. The wall was stitched with a simple, interrupted 2-0 absorbable monofilament suture (PDS II™, Ethicon Inc, Milan, Italy). The subcutis and skin were sutured with a continuous intradermal pattern, using 2-0 absorbable multifilament material (Vicryl™ Ethicon Inc, Milan, Italy).

### 2.7. Data Analysis

For group L, subjects requiring conversion from laparoscopic to traditional surgery were excluded for the statistical analyses. Data relating to weight, surgical time (from the first painful stimulus to the end of the surgical procedure), recovery time (all reflexes present and spontaneous movements after atipamezole administration), feeding time (from atipamezole administration to spontaneous feeding), time of release (from atipamezole administration to release into nature) were noted for both groups. Statistical analysis was performed using Microsoft Excel (Excel©, Microsoft, WA, USA). Comparisons between Groups were made using Student’s *t*-test, with a significance level of *p* < 0.05.

## 3. Results

Results regarding weight, surgical time, recovery time, feeding time and time of release are reported in Table 1.

In group T, salpingectomy was performed in *n* = 9 subjects, vasectomy in *n* = 17 subjects and hysterectomy in *n* = 18 subjects.

In group L, salpingectomy was performed in *n* = 8 subject and vasectomy in *n* =11 subjects.

During the post-operative captivation phase, none of the animals showed signs of apathy or anorexia.

Once released, all the animals quickly reached the water and started swimming.

Post-operative monitoring, during the days following surgery, was carried out via video footage and direct observations. No signs of infection, wound dehiscence, herniation, or ongoing pathology were noted. None of the nutrias showed changes in social behavior.

Statistical analysis with Student’s *t*-test did not show significant differences between the two groups, except for the surgical time, shorter for the traditional procedure (*p* = 0.004).

### Adverse Events

During the intraoperative period, there were no major surgical complications such as massive hemorrhages, accidental organ perforation, or cardiorespiratory arrest. Laparoscopy was converted to open surgery in *n* = 13 subjects. Moderate hemorrhage occurred during salpingectomy of *n* = 2 animals and, as a precaution, the procedure was converted after ablation of one salpinx.

In *n* = 9 subjects (*n* = 6 females and *n* = 3 males), an extremely replete gastrointestinal tract was found, not allowing correct visualization the reproductive tract, and surgery was converted. One subject was converted because of presence of parasalpingeal cysts. Another nutria (male) was converted due to malfunction of the laparoscopic insufflator.

In the days following the release, *n* = 2 subjects (*n* =1 male and *n* =1 female) which underwent laparotomic surgery (group T) were found dead. The necropsy highlighted the presence of extensive peritonitis.

## 4. Discussion

Animal reproduction control methods can be reversible or permanent. The former usually concern the modification of hormonal patterns. However, the effectiveness of these methods is based on repeated and regular administration of specific drugs, which is economically unsustainable and impractical in wildlife [11].

Permanent methods of reproductive control rely on surgical sterilization. Gonadectomy could represent a valid alternative, but negative effects on social behavior have been demonstrated in various species including rodents [12,23,24]. However, studies correlating gonadectomy with changes in social behavior in free-ranging nutrias colonies are lacking. Therefore, preserving the gonads with methods such as salpingectomy and vasectomy is more suitable for animals with complex hierarchical social structures [10,25].

The adult animals included in this study, both pregnant and not pregnant, had a higher mean weight than wild animals in their native area [26]; this is probably related to the anthropization of the subjects, which are fed daily by the citizens.

Traditional surgery was faster than laparoscopic surgery, as has been reported in several studies in human and veterinary medicine [27,28,29]. A possible cause for longer times for laparoscopy could be related to the acquisition of specific manual skills with a longer learning curve and greater training than traditional surgery. The anatomical differences between species in veterinary medicine make the acquisition of manual skills more complex, leading to an increase in surgical times and explaining the statistically significant difference detected (*p* < 0.05). Laparoscopic surgery has been described in mammals as rats, guinea pigs, rabbits, and capybaras [30,31,32,33,34], but no reports regarding nutrias were found; a comparison between laparoscopic vs. laparotomic ovariectomy (mean surgical time: 43 min vs. 22 min) was carried out in rabbits [28] with results comparable to the present study. The insufflation of the abdomen and the positioning of the trocars require a certain amount of time, spared if an open technique is used. On the other hand, laparoscopy makes it possible to gain minutes for the closure of the incisions, usually smaller.

The number of laparoscopic surgeries could have been higher if some young subjects, weighing less than 1000 g (*n* = 5 males and *n* = 4 females), had not been excluded from group L, assuming that the surgical set in use could not be suitable for such patients.

The laparoscopic technique (three-port technique and 5 mm laparoscopic instruments) performed is similar to procedures reported in other wild species [21,35]. Trendelenburg positioning, as recommended for reproductive tract surgery in rodents [36], ensured cranial displacement of the gastrointestinal tract allowing visualization of both salpinges and vasa deferentia and easy surgical resection of the reproductive tracts during laparoscopic procedures.

The apparently high number of conversions to traditional surgery (*n* = 9 subjects, 28.1%) is related to excessive gastro-enteric repletion due to the use of cage traps abundantly baited with food. To reduce the stress related to captivation times, the surgeries were performed shortly after capture and, therefore, it was not possible to fast the animals.

A conversion linked to malfunctioning of one of the components of the laparoscopic equipment (*n* = 1 equal, 3.1%) is related to the need to work in field settings since, most likely, when working in the surgical theatre, it would have been possible to carry out minimally invasive surgery, despite any malfunctioning.

Nutrias, like most rodents, have a voluminous caecum [37] but, nonetheless, perforation of the gastrointestinal tract did not occur during placement of the Veress needle and operating ports [38]. No further severe complication, such as rupture of large vessels or bladder, often reported in literature, occurred during these maneuvers [38].

Abdominal pressure was maintained at 8 mmHg, as described for laparoscopy in dogs [39,40] and rabbits [28]. This pressure allowed good visualization and maneuvering space for the instruments and did not cause any cardiorespiratory complications.

The use of an electrosurgical device (Onemytis^®^), exploiting Airplasma^®^ technology, was a valid aid in reducing complications such as hemorrhage and excessive tissue damage. This technology, in fact, uses the airborne plasma as a conductor of electricity, allowing adequate coagulation and ablation while maintaining a low tissue dissipation temperature of about 50 °C [41,42]. Onemytis^®^, never previously used in laparoscopy, has been proven to be effective in sealing the salpinges, the vasa deferentia and the relative vessels.

The choice to perform hysterectomy in pregnant subjects was dictated by an evaluation of the surgical risk, preferring a guaranteed hemostasis by ligation of the great vessels, rather than by electrosurgical devices [38]. The high number of pregnant females was probably related to the urbanization of subjects and their overfeeding, which allowed all pubescent females to carry on the pregnancy and to maintain a continuous reproductive cycle during the year, without hierarchic interferences [43].

The two-layer suture in a continuous intradermal pattern with sunken knots, in both types of surgeries, avoided the self-removal of the stitches, allowing rapid creation of epithelial bridges: none of the subjects showed signs of wound dehiscence in the days following surgery. This type of suture minimizes the risk of dehiscence following scratching or licking and, also, causes little tissue irritation as the suture material is not exposed to the external environment [44]. This is particularly important when dealing with wild animals that cannot be strictly monitored after release.

Mean time of spontaneous feeding in group L was slightly higher than in group T, although the difference was not significant. This difference could be related to a temporary decrease in gastro-intestinal motility, linked to the insufflation pressure during the pneumoperitoneum phase on replete organs, as reported in other hystricomorphic rodents such as guinea pigs [45].

As local legislation did not permit holding the subjects in captivity, even for relatively short times, a close follow-up of the animals was not possible in the immediate post-operative period. However, there were two cases of re-capture: in these nutrias, correct wound healing and no signs of infection or complication (hernia swelling, bruising, bruising, or pain) were noted.

The finding of two dead subjects belonging to group T in the days following the operations suggests that the risk of post-operative complications was higher in group T than in group L, despite the high professional level and technical ability of the surgeon. The deaths, in fact, occurred despite the great attention paid during the procedures, producing a very small-sized surgical wound, carefully sutured to ensure perfect repair of the layers. Nevertheless, since no overt pathological findings were noted during the intraoperative period, it is assumed that the origin of the peritonitis, in the dead subjects, could be directly related to the laparotomic procedure, or to the possible subsequent contamination of the surgical wound by water or solid material, due to the semiaquatic habits of the nutrias. Such complications are certainly less likely when laparoscopic surgery is performed, since the surgical wounds are reduced compared to laparotomic ones.

The follow-up, therefore, was limited to remote observation of the animals, in the days following the interventions, in their natural habitat, with no overt behavioral or postural changes that could suggest pain or discomfort.

The low rate of complications, the absence of anomalous behaviors and the rapid resumption of spontaneous feeding indicate that nutria subjected to these surgical techniques could be rapidly released on the territory, avoiding alterations to social hierarchies, and guaranteeing animal welfare in compliance with current regulations.

The results demonstrate that salpingectomy and vasectomy techniques represent an effective and safe method for the management of nutrias colonies in urban areas. Possible long-term medical complications of this type of surgeries, even on other animals, have not been well studied. However, it is expected that there will be no differences in terms of consequences on the health status of the animals, compared to intact subjects, as reported in dogs [46].

Laparoscopic salpingectomy could also be applicable in other female rodents for wildlife reproduction control; however, the technique employed could be adjusted according to the peculiar anatomical features of the considered species. For example, a bilateral laparoscopic approach has been proposed for other hystricomorphic rodents such as capybaras [21]. On the other hand, laparoscopic vasectomy could be an appropriate technique in other male rodents; in these animals, in fact, a surgical abdominal approach to the gonads is possible, as the presence of a functional cremaster and open inguinal canals allows the migration of the testicles into and out of the abdomen [47].

The procedures described in this work made it possible to comply with local legislation for the control of nutrias, avoiding euthanasia. This strategy can be successfully used in control plans to be implemented in urban environments where contacts and relationships with invasive alien species are common and public opinion have a considerable role [6].

It is reasonable that, even in this species, the reduced pain and injury caused by laparoscopic techniques would make this method preferable, despite the significantly longer execution times compared to traditional surgery. Undoubtedly, the logistical organization in the field and the cost of the procedures and equipment are certainly not comparable to those of euthanasia. However, the excellent results of the method used to numerically control limited animal nuclei in urban environment to suggest that this method could be applicable for the containment of nutrias.

## 5. Conclusions

The outcome of the developed and evaluated procedures suggests that laparoscopic salpingectomy and vasectomy represent an effective, safe, and versatile tool for the management of invasive alien species, particularly where the spread of the species occurs in urban areas, where application of other control methods could be hindered by public opinion and by associations fighting for animal rights.

## Figures and Tables

**Table 1:** Results regarding weight, surgical time, recovery time, feeding time and time of release in Group L and Group T, reported as Mean ± SD.

Variable	Group L	Group T
Weight (kg)	3.79 ± 1.57	4.23 ± 2.37
Surgical time (min)	28.4 ± 15.1	16.7 ± 4.7
Recovery time (min)	8.8 ± 4.6	9.6 ± 7.9
Feeding time (min)	28.1 ± 18.3	25.5 ± 18.7
Time of release (min)	371.1 ± 146.3	344.6 ± 169.2

## Data Availability

Further specific data regarding each animal can be requested to the Authors.
